# Hepcidin, in contrast to heparin binding protein, does not portend acute kidney injury in patients with community acquired septic shock

**DOI:** 10.1371/journal.pone.0299257

**Published:** 2024-05-02

**Authors:** Jon Olinder, Matilda Jovanovic Stjernqvist, Albin Lindén, Evelina Thaphikul Salomonsson, Martin Annborn, Heiko Herwald, Cecilia Rydén

**Affiliations:** 1 Department of Clinical Sciences, Division of Infection Medicine, Lund University, Lund, Sweden; 2 Department of Clinical Sciences, Sections of Anesthesiology and Intensive Care, Lund University, Lund, Sweden; 3 Department of Anesthesiology and Intensive Care, Helsingborg Hospital, Helsingborg, Sweden; Pelita Harapan University Faculty of Medicine: Universitas Pelita Harapan Fakultas Kedokteran, INDONESIA

## Abstract

**Background:**

Acute kidney injury (AKI) is a common and severe complication in patients treated at an Intensive Care Unit (ICU). The pathogenesis of AKI has been reported to involve hypoperfusion, diminished oxygenation, systemic inflammation, and damage by increased intracellular iron concentration. Hepcidin, a regulator of iron metabolism, has been shown to be associated with sepsis and septic shock, conditions that can result in AKI. Heparin binding protein (HBP) has been reported to be associated with sepsis and AKI. The aim of the present study was to compare serum hepcidin and heparin binding protein (HBP) levels in relation to AKI in patients admitted to the ICU.

**Methods:**

One hundred and forty patients with community acquired illness admitted to the ICU within 24 hours after first arrival to the hospital were included in the study. Eighty five of these patients were diagnosed with sepsis and 55 with other severe non-septic conditions. Logistic and linear regression models were created to evaluate possible correlations between circulating hepcidin and heparin-binding protein (HBP), stage 2–3 AKI, peak serum creatinine levels, and the need for renal replacement therapy (RRT).

**Results:**

During the 7-day study period, 52% of the 85 sepsis and 33% of the 55 non-sepsis patients had been diagnosed with AKI stage 2–3 already at inclusion. The need for RRT was 20% and 15%, respectively, in the groups. Hepcidin levels at admission were significantly higher in the sepsis group compared to the non-sepsis group but these levels did not significantly correlate to the development of stage 2–3 AKI in the sepsis group (p = 0.189) nor in the non-sepsis group (p = 0.910). No significant correlation between hepcidin and peak creatinine levels, nor with the need for RRT was observed. Stage 2–3 AKI correlated, as expected, significantly with HBP levels at admission in both groups (Odds Ratio 1.008 (CI 1.003–1.014, p = 0.005), the need for RRT, as well as with peak creatinine in septic patients.

**Conclusion:**

Initial serum hepcidin, and HBP levels in patients admitted to the ICU are biomarkers for septic shock but in contrast to HBP, hepcidin does not portend progression of disease into AKI or a later need for RRT. Since hepcidin is a key regulator of iron metabolism our present data do not support a decisive role of initial iron levels in the progression of septic shock into AKI.

## Introduction

Sepsis and septic shock are consequences of a dysregulated host response to infection often complicated by acute kidney injury (AKI) [1–3]. AKI is a frequent organ dysfunction acquired by many patients in need of intensive care [2,3]. Sepsis-associated acute kidney injury (S-AKI) has reported mortality rates of up to 46–65% [2,3]. Furthermore, among survivors of S-AKI the risk of progression to chronic kidney disease (CKD) is substantial [4]. The pathogenesis of S-AKI is multifactorial and reportedly involves hypoperfusion, aberrant cytokine stimulation but also the formation of reactive oxygen species (ROS) [5,6]. Iron overload can lead to AKI due to ROS-induced lipid peroxidation and iron deposition in kidney cells resulting in cell death through the process of ferroptosis [7,8]. High levels of circulating iron have been shown to be associated with renal ischemia-reperfusion injury (IRI) that contributes to AKI [9–12].

The 25-amino-acid peptide hepcidin is an important regulator of circulating iron levels in normal homeostasis, as well as in anemias including those associated with inflammation [13,14]. Hepcidin inhibits intestinal iron uptake and downregulates ferroportin that transports iron from cells to the plasma [13,14]. Circulating hepcidin is an acute-phase reactant that is largely excreted by hepatocytes upon stimulation by IL-6 [15]. Hepcidin also exhibits antibacterial and antifungal activity [15–19]. An increased mortality has been reported for Intensive Care Unit (ICU) patients that require RRT and who exhibit low levels of circulating hepcidin [20]. This would suggest that hepcidin could be used as a prognostic biomarker for AKI and/or for the need of RRT [20]. Furthermore, data from animal experiments show that exogenously administrated hepcidin reduces the risk of ischemic renal injury (IRI) in lipopolysaccharide-induced sepsis [21]. A protective effect of hepcidin on hemoglobin-mediated kidney injury has been reported [22,23]. AKI is also a common complication in patients that have undergone cardio-pulmonary bypass (CPB) procedures [24,25]. Patients with high levels of urinary hepcidin have a reduced risk to acquire AKI after CPB [24–26]. The relevance of urinary hepcidin as to a specific protective effect on post-CPB AKI has, however, been disputed [27,28]. In patients with chronic kidney disease (CKD) it is reported that reduced renal clearance and inflammation increases hepcidin that in turn causes systemic iron-deficiency contributing to anemia of CKD [29].

Another potential marker for sepsis-induced AKI is heparin-binding protein (HBP) [30–32]. HBP, is a cationic antimicrobial protein produced in neutrophils and stored in both primary granules and secretory vesicles, and is rapidly released *e*.*g*. upon bacterial phagocytosis [33,34]. HBP influences vascular permeability and acts as a chemoattractant for cells [34–36]. Several studies have shown that HBP is a promising biomarker for sepsis and circulatory failure and correlates with the development of AKI in septic subjects [30–32].

The aim of the present study was to explore the associations between hepcidin and HBP levels in serum and renal failure among critically ill patients with sepsis or non-septic conditions. The investigated, well-defined cohort consisted of patients with community acquired illness who had to be transferred to intensive care within 24 hours after their first admission to the hospital. Upon admission to the ICU, serum levels of hepcidin and HBP were recorded and investigated as to their correlation with later development of AKI stage 2–3, peak creatinine, and need of renal replacement therapy in septic and non-septic patients.

## Methods

### Study design

This prospective observational study was conducted at the ICU in Helsingborg, Sweden from 1^st^ of May 2014 until 31^st^ of August 2020. No patients suffering from Sars-Cov-2 were included. The methods used in the study were performed according to relevant regulations and guidelines.

The study was approved by the Ethics Committee, University of Lund, Sweden (Approval nb. 2014/4, 2014/195, 2015/467, 2019/04558). Informed consent was collected from the patient or next of kin, the latter if the patient was unable to confirm upon inclusion. Delayed consent from the patient was accepted by the Ethics Committee.

#### Patient population and definitions

The patient cohort investigated in the present study has been described previously [37]. The sepsis patients were enrolled in line with the sepsis-3 definition–anamnesis/history of infection and SOFA score ≥ 2, and the patients in the non-sepsis group were admitted with totally different diagnosis, *e*.*g*. trauma, myocardial infarction, or epilepsy. Patients were included consecutively by the ICU physicians, according to inclusion criteria as well as considering current work load in the ICU. In the present study investigating AKI 24 patients presenting with chronic kidney disease (CKD) were excluded, as well as 14 patients lacking admittance samples for hepcidin and/or HBP. In short, patients above the age of 18 years who were transferred to the ICU within 24 hours of hospital admission and with a suspected stay in the ICU for >3 days, without CKD were included in the study. Patients who had a blood transfusion or surgery within the preceding 7 days prior to the admission or if the need for intensive care was expected to be less than 3 days. Blood cultures and urine cultures were performed at inclusion. Patients were not diagnosed or treated by the physicians involved in the study. The final evaluation of all data was assessed at the end of the study when patients had been categorized as having had either sepsis or a non-septic condition. Sepsis was defined as a proven or highly suspected infection with a Sequential Organ Failure Assessment (SOFA) score ≥ 2 according to the Sepsis-3 criteria [1]. Patients included before the 2016 sepsis-3 criteria were implemented, were evaluated specifically to assure that they also fulfilled the updated Sepsis-3 criteria. Established ICU scoring systems *i*.*e*., Simplified Acute Physiology Score 3 (SAPS III) and SOFA score were evaluated at admission and SOFA scores were registered every day during the study period. The need of intubation and vasopressor support were registered as well as the need of renal replacement therapy (RRT) by hemodialysis (either intermittent or continuous) during the ICU-stay. The classifications of Sepsis-3 and Kidney Disease Improving Global Outcomes (KDIGO) in our cohort was up to date with the literature and clinical practice [38].

As described in the KDIGO guidelines the criteria for AKI stage 1 include increased serum creatinine ≥ 1.5-fold baseline within the last 7 days, or an increase of serum creatinine (SCr) by 0.3 mg/dL (≥26.5 μmol/L) in the last 48 hours, or a urine volume of <0.5 mL/kg/h for at least 6–12 hours. An increased SCr of 2.0–2.9 times baseline value, or if urine output of ≤0.5 mL/kg/h lasts for 12–24 hours, the criteria for AKI stage 2 have been fulfilled. The most severe form of AKI, stage 3, is diagnosed through either a ≥3-fold increase in serum creatinine compared to baseline, a SCr of 4.0 mg/dL (353.6 μmol/L), or the initiation of renal replacement therapy (RRT).

Demographics, co-morbidities and 28-day mortality were registered. In the group with non-septic conditions the primary organ dysfunction causing the enrolment was registered.

### Analysis of biomarkers

Blood samples for the analysis of hepcidin, HBP, and creatinine concentration, were collected at the time of inclusion and the clinical status and laboratory parameters of all patients were followed for seven consecutive days in the ICU. Hepcidin was analyzed at the Clinical Chemistry Laboratory, Lund University Hospital, Sweden, using Mass Spectrometry with a 6500 Q-trap, Sciex, Washington D.C., USA [39]. HBP was analyzed with an in-house enzyme-linked immunosorbent assay (ELISA) method developed at the Department of Clinical Sciences, Lund, Division of Infection Medicine, Biomedical Center at Lund University, Sweden [40]. Creatinine was analyzed with the Sysmex XE-550 system at Helsingborg Hospital, Sweden. Microbial analyses were performed at the Clinical Microbiological Laboratory at Lund University Hospital, Sweden. Blood cultures as well as cultures from other adequate locations, *e*.*g*. wounds, urine, sputum, and nasopharynx, were obtained at inclusion before antibiotic treatment was initiated, and repeated according to the progression of the patients’ illness as judged by the physician in charge of the patient.

### Acute kidney injury

The study population was divided into AKI stage 2–3, if the peak value of serum creatinine (SCr), exceeded 2 times the baseline SCr, or if RRT was needed, or no kidney failure *i*.*e*. AKI 0, or AKI stage 1 if none of the abovementioned requirements were met according to the KDIGO guidelines [38]. Values of baseline SCr were obtained from patients’ medical records. If no baseline SCr was available, the baseline creatinine level was calculated according to the recommendations by the Acute Dialysis Quality Initiative (ADQI) using the Modification of Diet in Renal Diseases (MDRD) equation, assuming a GFR of 75ml/min/1,73m^2^ [41,42].

Patients were dichotomized into only two subgroups due to very small differences in creatinine levels between patients with AKI 0, *i*.*e*. no impact on kidney function, and AKI stage 1, 2 and 3, respectively. The decision to use the creatinine as criterium to categorize AKI was made according to previous reports emphasizing missing registration of fluid intake and urine output [43].

### Statistics

All data were analyzed in IBM SPSS version 28. Patients with CKD and patients with missing values of hepcidin or HBP at admission to the ICU were excluded from the analyses. Mann Whitney U-test was used to compare medians. Pearson’s Chi-Squared test was performed to investigate differences in 28-day mortality between the groups (sepsis and non-sepsis). Regression models were created to examine the effects of either hepcidin or HBP on the development of AKI stage 2–3, the need for renal replacement therapy, and peak serum creatinine concentrations, respectively. Concentrations of hepcidin and HBP at admission, age, and sex were used as independent variables in sepsis versus non-sepsis patients. Additionally, if significant results were achieved in the regression models, the model was expanded with an interaction variable to evaluate and adjust for the possible interaction between sepsis and hepcidin or HBP. An adjusted (by age and sex) logistic regression model was created to examine the association between serum concentrations of hepcidin and HBP, respectively, and AKI stage 2–3. A linear regression model was created to estimate if hepcidin or HBP correlated with peak creatinine level. A bivariate regression model was created using admission values of hepcidin and HBP, respectively, to analyze potential correlations between these biomarkers with the requirement of dialysis. Due to the limited number of patients requiring RRT, values of hepcidin and HBP, respectively, and sepsis were used as independent variables (n = 25). A confidence interval (CI) of 95% and a p-value ≤ 0.05 were considered significant for all statistical analyses.

## Results

**Fig 1:**
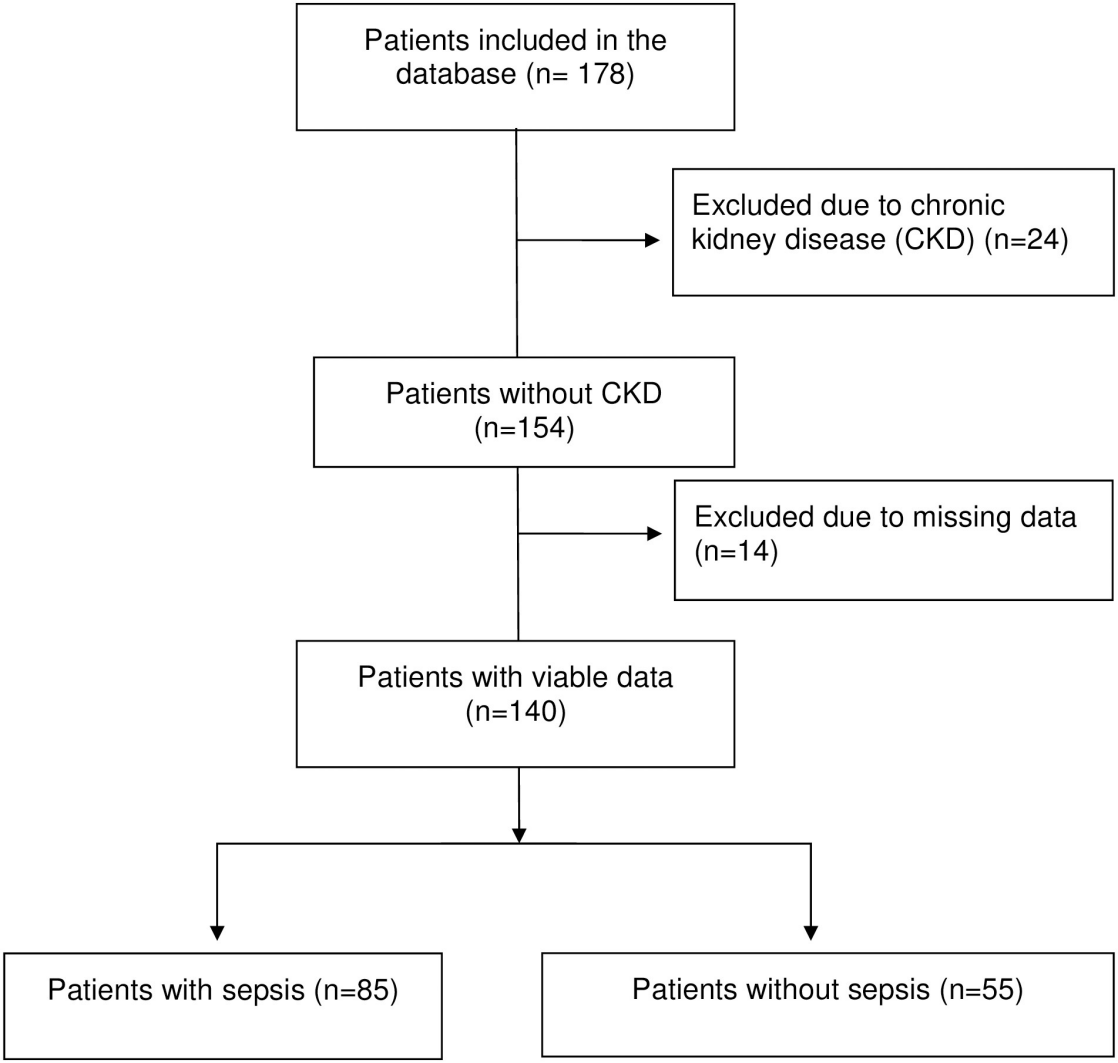
Patient flowchart.

A total of 178 patients were included in the study, 24 were excluded due to a CKD diagnosis, and 14 due to missing hepcidin or HBP values on the day of admission (Fig 1). Of the remaining 140 patients, 85 were diagnosed with sepsis (83 fulfilled the updated Sepsis-3 criteria of septic shock, the remaining two needed ICU surveillance and non-invasive ventilation treatment), and 55 patients were admitted with non-infectious conditions including cardiovascular events such as acute myocardial infarction and severe heart failure, trauma, *e*.*g*. car accidents and fall accidents, abdominal illness, *e*.*g*. ileus, neurological, *e*.*g*. status epilepticus, respiratory failure due to *e*.*g*. aspiration, and metabolic *e*.*g*. severe electrolyte impairment (Table 1). Most patients developed prerenal kidney failure, with 98% of sepsis patients in need of vasopressor support to maintain blood pressure already at inclusion. In comparison 81% of the non-sepsis patients needed vasopressor support to maintain blood pressure, where a prerenal component was most common (Table 1).

**Table 1:** Demographics of patients.

Characteristics	Sepsis	Non-sepsis
Patients included	85 (61%)	55 (39%)
Male/female %	56/44	62/38
Median Age (IQR)	70 (57–76)	69 (53–74)
**Comorbidity**		
Cardiovascular disease	38 (45%)	22 (40%)
Hypertension	25 (29%)	20 (36%)
Diabetes	21 (25%)	13 (24%)
COPD	13 (15%)	14 (26%)
Malignancy	10 (12%)	6 (11%)
Liver disease	5 (6%)	0 (0%)
**Source of infection**		
Respiratory	38 (45%)	-
Urogenital	16 (19%)	-
Skin/soft tissue	14 (17%)	-
Abdominal	8 (9%)	-
Unknown source	8 (9%)	-
Endocarditis	1 (1%)	-
**Condition (non-sepsis patients)**		
Cardiovascular	-	19 (35%)
Trauma	-	10 (18%)
Abdominal	-	10 (18%)
Neurological	-	7 (13%)
Respiratory	-	3 (6%)
Metabolic	-	2 (4%)
Unknown	-	2 (4%)
Renal	-	1 (2%)
Intoxication	-	1 (2%)
**ICU-data**		
SOFA-score at admission	11 (IQR 7–12)	9 (IQR 7–10)
SAPS III score	67 (IQR 60–74)	65 (IQR 54–79)
Intubation	45 (53%)	50 (91%)
Vasopressor support	83 (98%)	45 (82%)
Renal replacement therapy	17 (20%)	8 (15%)

IQR, interquartile range. COPD, chronic obstructive pulmonary disease.

In 27/85 sepsis patients and 12/55 non-sepsis patients baseline creatinine was not available at inclusion, and therefore calculated using the abovementioned MDRD equation, assuming a GFR of 75ml/min/1,73m^2^. None of these patients without previous baseline creatinine at inclusion had an underlying history or diagnosis of CKD. The calculated median creatinine levels at arrival to the hospital of the patients where baseline creatinine was not available, were 88.0 μmol/L (IQR 80.0–97.0) in sepsis and 71.0 μmol/L (IQR 71.0–94.8) in non-sepsis patients, respectively. Among the 140 patients evaluated, 44 patients with sepsis and 18 with a non-septic condition, met the criteria of either a ≥ two-fold increase in serum creatinine compared to baseline level, or a need for renal replacement therapy during the period of intensive care, corresponding to AKI stages 2–3 (Table 2). The median AKI stage was statistically higher in the sepsis group compared to the non-sepsis group upon admission (p = 0.031), (Table 2). The number of patients, hepcidin, HBP, and creatinine levels upon admission for sepsis and non-sepsis patients, respectively in each AKI-stage (0–3) are all presented in Table 3a and 3b.

**Table 2:** Acute kidney injury stage 0–3 in sepsis and non-sepsis patients, respectively. Number of patients with sepsis and non-sepsis in each AKI group, % of patients within each group. Stage difference between sepsis and non-sepsis patients calculated with Mann-Whitney U-test.

Acute Kidney Injury, (AKI) stage	Sepsis n = 85	Non-sepsis n = 55	p-value
AKI-stage 0–3, median (IQR)	2 (0–3)	1 (0–2)	p = 0.031
AKI-stage 0	22 (26%)	23 (42%)	
AKI-stage 1	19 (22%)	14 (26%)	
AKI-stage 2	15 (18%)	5 (9%)	
AKI-stage 3	29 (34%)	13 (24%)	

IQR = interquartile range. AKI-stage 0 is equivalent to no kidney failure.

**Table 3:** a. Median hepcidin, HBP, and creatinine values at admission, respectively, in patients with Acute Kidney Injury (AKI) stage 0–3 in sepsis patients. IQR = interquartile range. AKI-stage 0 is equivalent to no kidney failure. b. Median hepcidin, HBP, and creatinine values at admission, respectively, in patients with Acute Kidney Injury (AKI) stage 0–3 in non-sepsis patients. IQR = interquartile range. AKI-stage 0 is equivalent to no kidney failure.

**AKI stage**	**Sepsis** **n = 85**	**Hepcidin nmol/L**	**HBP** **ng/mL**	**Creatinine** **μmol/L**
AKI-0 (IQR)	22	36.5 (18.8–67.3)	34.7 (20.7–64.0)	78.5 (64.8–91.3)
AKI-1 (IQR)	9	41.0 (25.0–62.0)	38.2 (32.2–65.4)	128.0 (107.0–155.0)
AKI-2 (IQR)	15	48.0 (21.0–70.0)	55.5 (34.7–85.6)	151.0 (127.0–170.0)
AKI-3 (IQR)	29	56.0 (24.0–71.5)	86.3 (31.0–438.0)	229.0 (184.5–345.0)
**AKI stage**	**Non-Sepsis** **n = 55**	**Hepcidin nmol/L**	**HBP** **ng/mL**	**Creatinine** **μmol/L**
AKI-0 (IQR)	23	16.0 (6.5–39.0)	13.7 (12.2–20.0)	79.0 (55.0–92.0)
AKI-1 (IQR)	14	13.5 (6.7–28.3)	33.3 (20.2–54.8)	117.5 (101.8–138.8)
AKI-2 (IQR)	5	4.0 (0.8–31.5)	42.6 (21.0–187.6)	183.0 (145.5–191.0)
AKI-3 (IQR)	13	15.0 (1.4–41.0)	36.9 (24.3–50.6)	285.0 (187.5–323.5)

At study inclusion the median levels of hemoglobin (Hb) were 120 g/L (IQR 106.0–130.8) for males and 108 g/L (IQR 97.5–123.2) for females in the septic group. Corresponding values for hemoglobin between the genders in the non-septic group were 119 g/L (IQR 104.5–139.3) and 118 g/L (IQR 94.3–124.8), respectively. According to the World Health Organization (WHO) anemia is defined as Hb levels < 130 g/L in men and < 120 g/L in women [44].

### Serum hepcidin and HBP levels at admission

**Fig 2:**
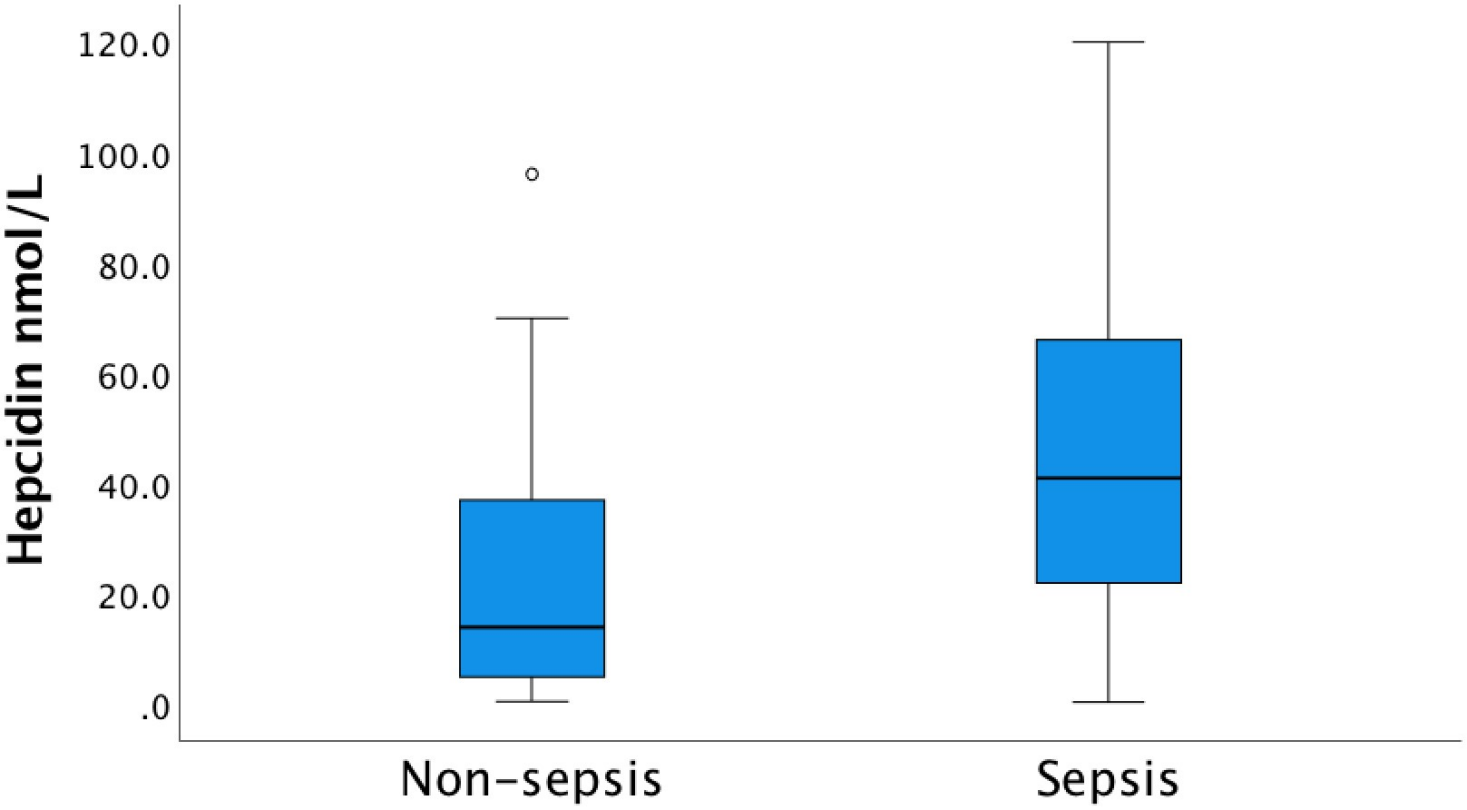
Concentration of hepcidin at admission to the ICU among sepsis and non-sepsis patients respectively. The median hepcidin concentration in the sepsis group (41.0 nmol/L, IQR 21.5–66.5) was significantly higher than in the non-sepsis group (14.0 nmol/L, IQR 4.2–37.0), p<0.001.

**Fig 3:**
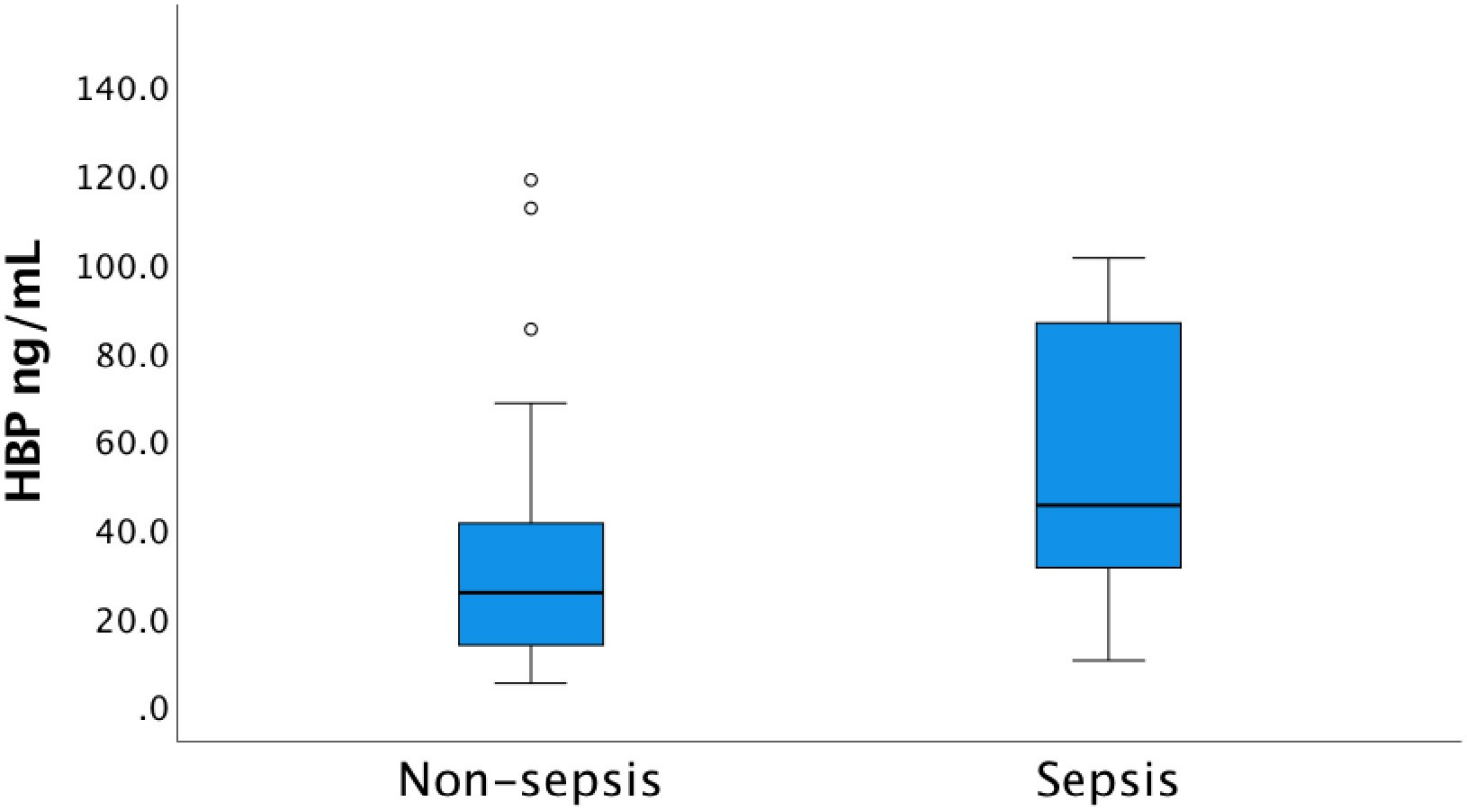
Concentration of heparin-binding protein (HBP) at admission to the ICU among sepsis and non-sepsis patients respectively. The median HBP concentration in the sepsis group (45.2 ng/mL, IQR 31.0–93.7) was significantly higher than in the non-sepsis group (25.3 ng/mL, IQR 13.5–42.6), p<0.001. In the graph, 12 extreme outliers and 6 outliers in the sepsis group as well as one extreme outlier in the non-sepsis group are excluded to enhance the readability. These patients were, however, included in the statistics.

In agreement with our earlier reported data, significantly increased levels of both hepcidin and HBP were recorded in the sepsis group compared to the non-sepsis group [37]. Median levels of hepcidin in the sepsis group at inclusion was 41.0 nmol/L, (IQR 21.5–66.5), compared to 14.0 nmol/L (IQR 4.2–37.0) in the non-sepsis group (p<0.001) (Fig 2). Corresponding levels of HBP at admission were 45.2 ng/mL (IQR 31.0–93.7) in the sepsis group versus 25.3 ng/mL (IQR 13.5–42.6) in the non-sepsis group (p = 0.002) (Fig 3). The patient cohort investigated herein does not include patients with CKD but were extracted from the same cohort as described by Olinder *et al*. where analysis of kidney failure was not reported [37]. The omission of patients with CKD, did not change the overall differences in hepcidin or HBP levels between sepsis and non-sepsis patients.

### Levels of serum hepcidin and HBP in patients with acute kidney injury

**Fig 4:**
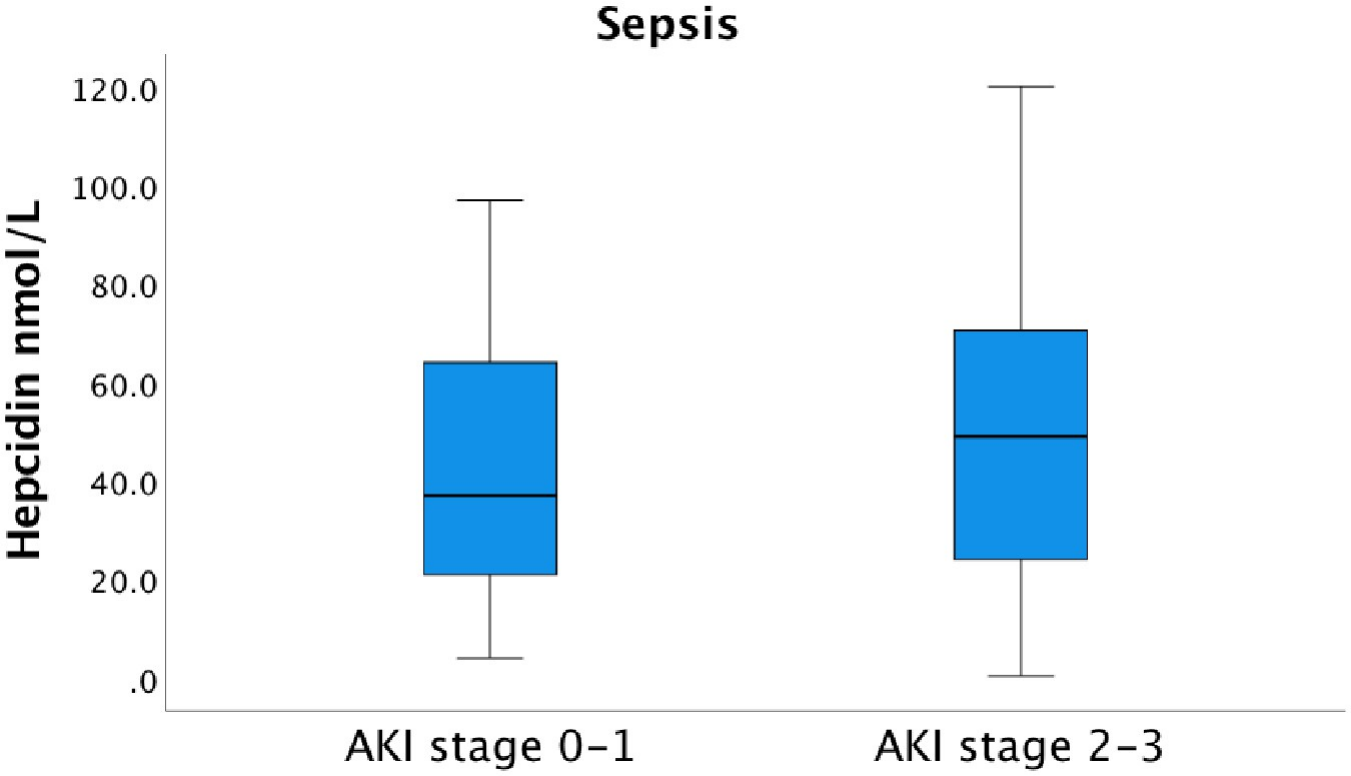
Serum concentration of hepcidin and the AKI stage among the sepsis patients at admission to the ICU. The median hepcidin serum concentration upon arrival to the ICU was 37.0 nmol/L (IQR 20.5–64.0) in the AKI 0–1 group and 49.0 nmol/L (IQR 23.0–70.8) in the AKI 2–3 group, not significantly different, p = 0.239. AKI-stage 0 is equivalent to no kidney failure.

The patients in the sepsis group with AKI stage 2–3 showed a median hepcidin serum concentration of 49.0 nmol/L (IQR 23.0–70.8) versus 37.0 nmol/L (IQR 20.5–64.0) in sepsis patients with AKI 0 and AKI stage 1 (Fig 4).

**Fig 5:**
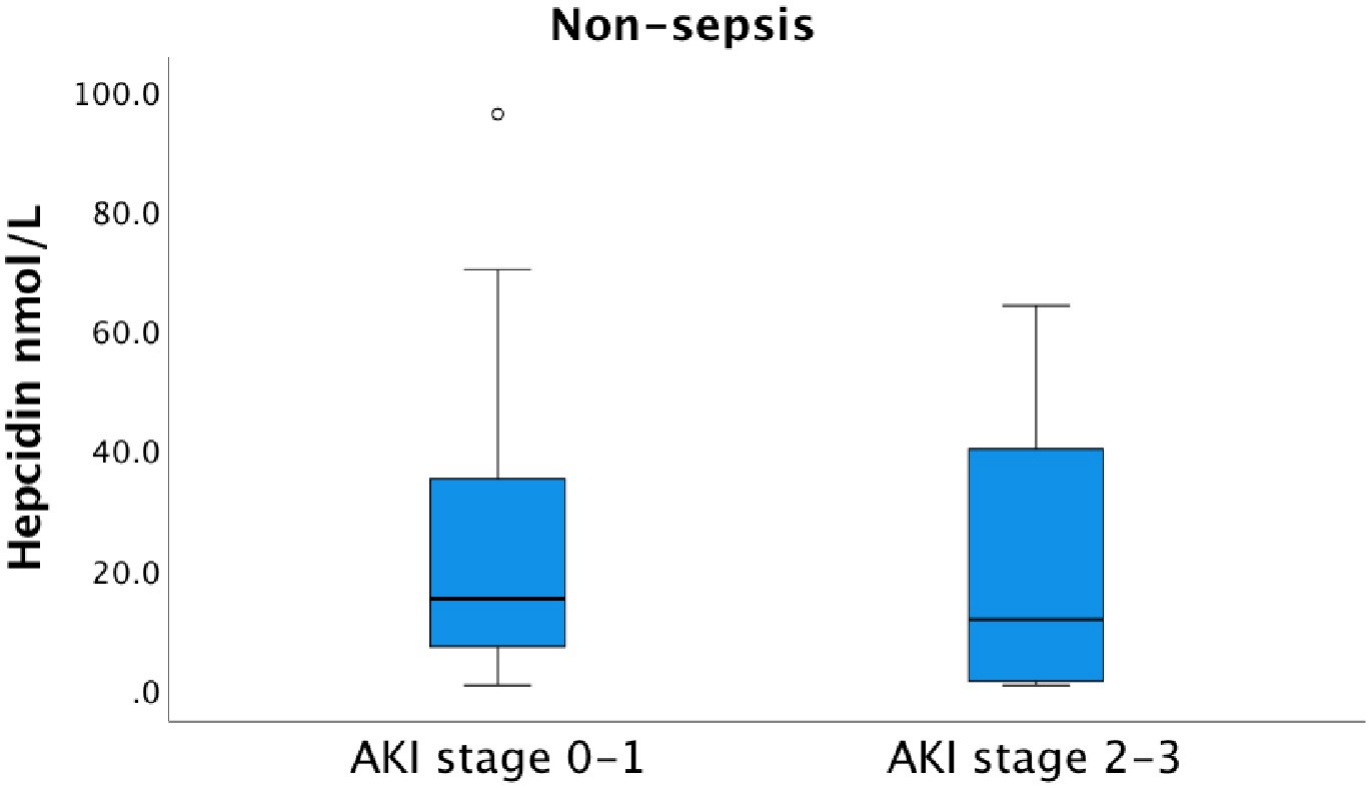
Serum concentration of hepcidin and the AKI stage among the non-sepsis patients at admission to the ICU. The median hepcidin serum concentration upon arrival to the ICU was 15.0 nmol/L (IQR 6.8–36.0) in the AKI 0–1 group and 11.5 nmol/L (IQR 1.2–40.5) in the AKI 2–3 group, not significantly different, p = 0.507. AKI-stage 0 is equivalent to no kidney failure.

In the non-sepsis group with AKI stage 2–3 the median hepcidin serum concentration was 11.5 nmol/L (IQR 1.2–40.5) versus 15.0 nmol/L (IQR 6.8–36.0) in non-septic patients with AKI 0 and AKI stage 1 (Fig 5).

No significant associations between hepcidin levels at admission and stage 2–3 AKI were observed in the sepsis group, Odds Ratio (OR) 1.011 (CI 0.995–1.027, p = 0.189), or in the non-sepsis group, OR 1.002 (CI 0.975–1.029, p = 0.910).

**Fig 6:**
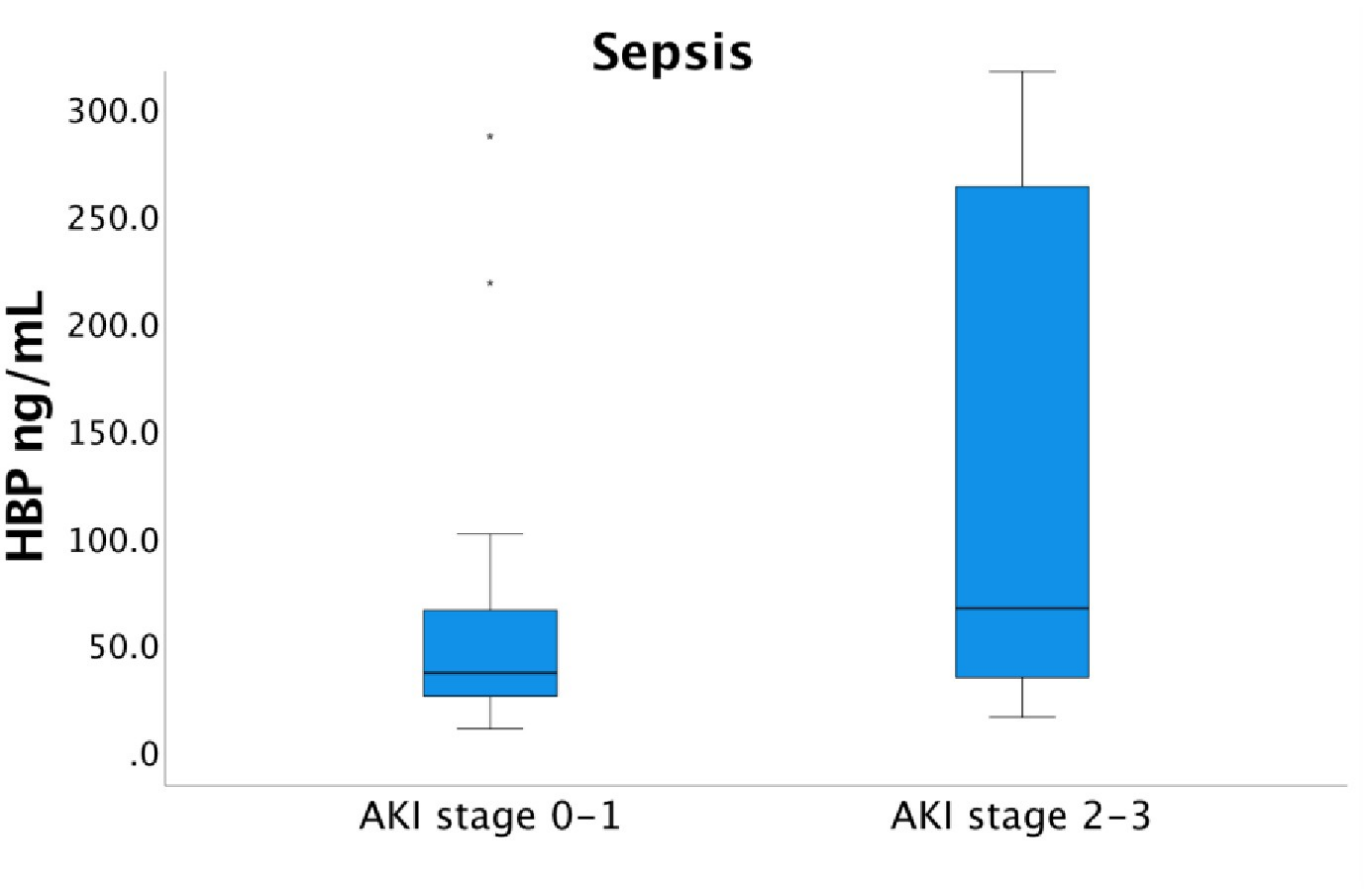
Serum concentration of HBP and the AKI stage among the sepsis patients at admission to the ICU. The median HBP serum concentration upon arrival to the ICU was 36.0 ng/mL (IQR 23.7–65.4) in the AKI 0–1 group and 66.3 ng/mL (IQR 33.8–265.4) in the AKI 2–3 group, significantly different, p = 0.002. In the graph, one extreme outlier in the AKI stage 0–1 group as well as two outliers and three extreme outliers in AKI stage 2–3 group are excluded to enhance the readability. These patients were, however, included in the statistics. AKI-stage 0 is equivalent to no kidney failure.

**Fig 7:**
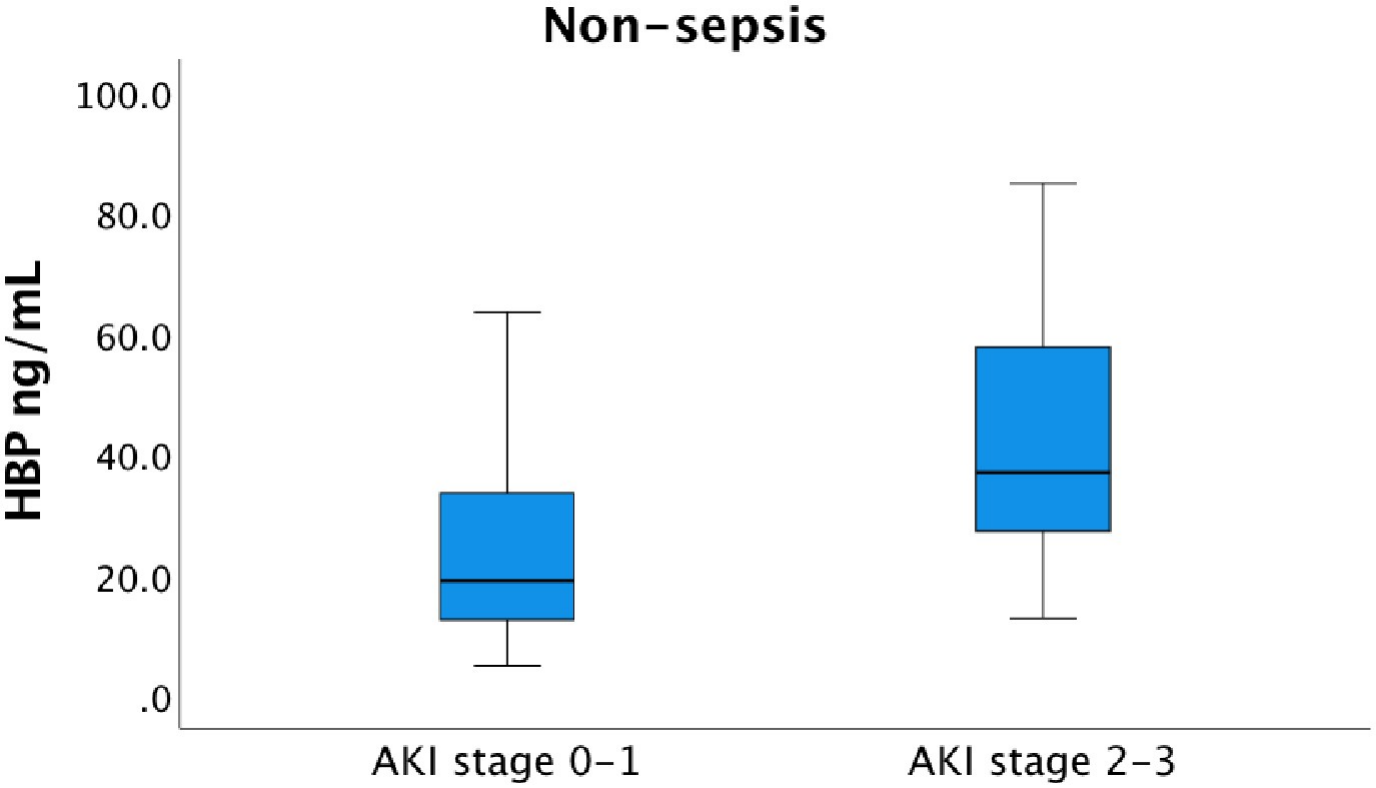
Serum concentration of HBP among the non-sepsis patients at admission to the ICU. The median HBP serum concentration upon arrival to the ICU was 19.0 ng/mL (IQR 12.5–34.6) in the AKI 0–1 group and 36.9 ng/mL (IQR 25.7–60.3) in the AKI 2–3 group, significantly different, p = 0.002. In the graph, one extreme outlier in the AKI stage 0–1 group as well as two outliers and three extreme outliers in AKI stage 2–3 group are excluded to enhance the readability. These patients were, however, included in the statistics. AKI-stage 0 is equivalent to no kidney failure.

There was a significant difference of median HBP serum concentrations upon arrival to the ICU between sepsis patients with AKI stage 2–3 and AKI 0 and AKI stage 1, 66.3 ng/mL (IQR 33.8–265.4) and 36.0 ng/mL (IQR 23.7–65.4) respectively, (p = 0.002) (Fig 6). Among the non-sepsis patients, the median HBP serum concentration was significantly higher, 36.9 ng/mL, in patients with AKI stage 2–3 versus 19.0 ng/mL (IQR 12.5–34.6) in patients with AKI 0 and AKI stage 1 group (p = 0.002) (Fig 7). Significant associations between HBP levels at admission and stage 2–3 AKI were observed both in the sepsis group with an OR 1.007 (CI 1.001–1.012, p = 0.012) and the non-sepsis group OR 1.033 (CI 1.005–1.060, p = 0.018). The analyses of HBP and AKI 2–3 were expanded with an interaction variable created to evaluate the possible interaction effect between sepsis and HBP. Differences concerning the associations between HBP and AKI in the sepsis- and non-sepsis groups analyzed together resulted in a p-value of 0.086. Finally, the regression model was utilized for the whole group (sepsis and non-sepsis) and a significant association between HBP and AKI stage 2–3 was observed, OR 1.008 (CI 1.003–1.014, p = 0.005).

### Hepcidin serum levels and renal replacement therapy

A total of 25 patients needed renal replacement therapy (RRT) (18%), 17 patients in the sepsis group (20%) and 8 in the non-sepsis group (15%). Median hepcidin levels on admission was 31.0 nmol/L (IQR 11.5–62.0) for patients across both groups requiring RRT compared to 34.0 nmol/L (IQR 13.0–59.0) for the non-RRT patients. In the septic group requiring RRT the median hepcidin serum concentration was 41.0 nmol/L (IQR 24.0–68.5) versus 11.5 nmol/L (IQR 0.8–28.5) in the non-septic group.

No significant associations were found between the hepcidin serum concentration upon admission and renal replacement therapy in either the sepsis group, OR 1.000 (CI 0.981–1.019, p = 0.999) or the non-sepsis group OR 0.991 (CI 0.949–1.035, p = 0.696).

### HBP serum levels and renal replacement therapy

For patients with sepsis requiring RRT, the median HBP level on admission was 268 ng/mL (IQR 29.3–738.7) compared to 36.4 ng/mL (IQR 23.5–41.8) for the non-septic patients.

Among septic patients, a significant association between the HBP serum concentration upon admission, and renal replacement therapy was found OR 1.008 (CI 1.003–1.012, p<0.001). In the non-sepsis group, no significant association was found between HBP upon admission and the need for RRT OR 1.001 (CI 0.982–1.020, p = 0.901), and the model was expanded with an interaction variable giving a p-value of 0.496. Finally, when including the whole group (sepsis and non-sepsis), a significant association between HBP at inclusion and the need for RRT was noted, OR 1.007 (CI 1.003–1.011, p = 0.001).

### Peak creatinine levels

**Fig 8:**
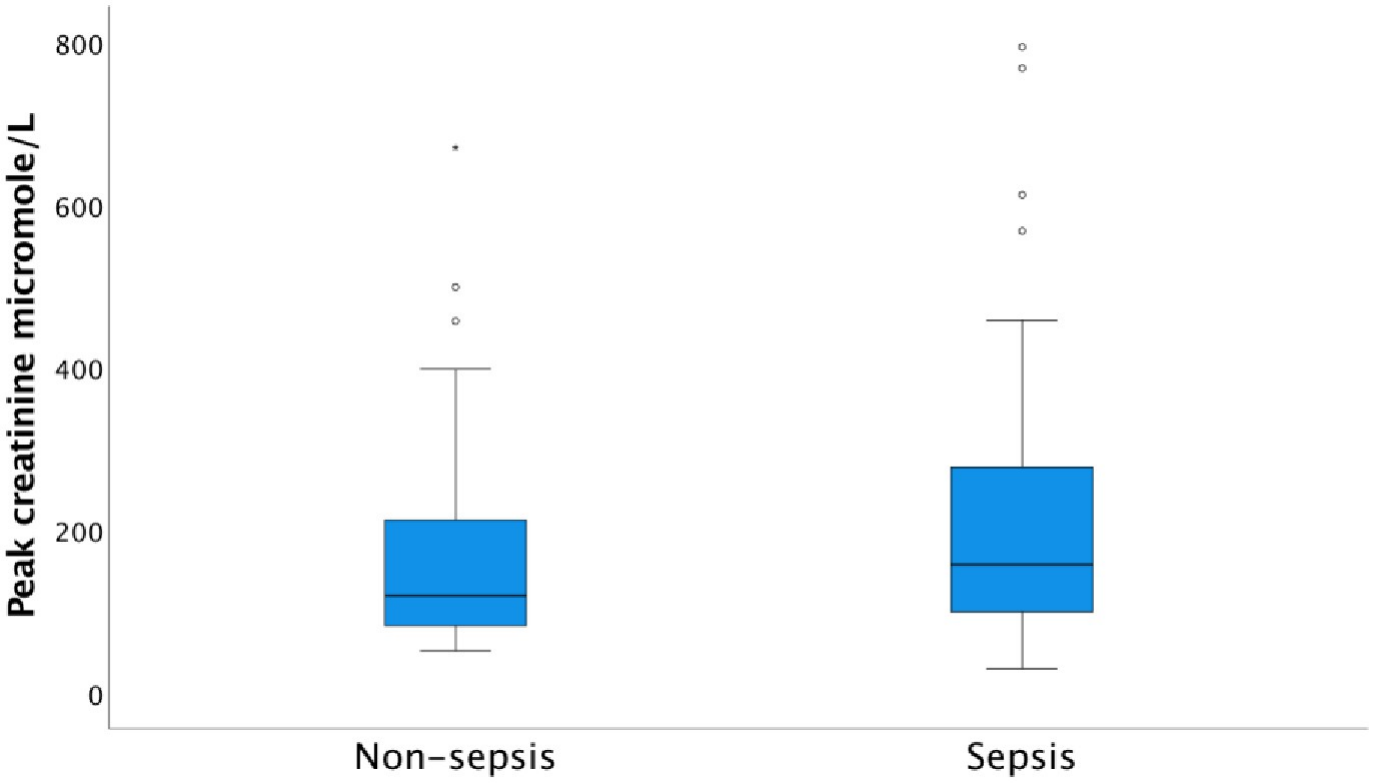
Median peak creatinine level at admission in non-sepsis versus sepsis patients. The median peak creatinine level was not significantly higher at admission in the sepsis group (157 μM/L) than in the non-sepsis group (119 μM/L), p = 0.085.

The median peak creatinine level at inclusion was 157.0 μmol/L (IQR 98.5–286.0) in the sepsis group and the corresponding value in the non-sepsis group was 119 μmol/L (IQR 81.0–215.0), (p = 0.119) (Fig 8).

For each increase of 1 nmol/l of hepcidin upon admission the corresponding difference in peak serum creatinine level (defined as B) were neither significant in the sepsis group, B = 0.637 μmol/L (CI -0.489–1.764, p = 0.264) nor in the non-sepsis group, B = 0.133 μmol/L (CI -1.525–1.791, p = 0.873).

In contrast with hepcidin, for each increase of 1 ng/mL of HBP upon admission the corresponding difference in peak serum creatinine level (defined as B), was significantly associated with peak creatinine values in the sepsis group, B = 0.190 μmol/L (CI 0.056–0.325, p = 0.006), whereas no significant correlation was observed between peak creatinine values and HBP levels in the non-sepsis group, B = 0.486 μmol/L (CI -0.452–1.423, p = 0.303). The analyses of sepsis and peak creatinine were expanded with an interaction variable created to evaluate the possible interaction effect between sepsis and HBP. Differences concerning the associations in the sepsis- and non-sepsis groups analyzed together resulted in a p-value of 0.569. The regression model was then utilized for the whole group (sepsis and non-sepsis) where a significant association between HBP and peak creatinine was observed, B = 0.210 μmol/L (CI 0.084–0.335, p = 0.001).

### Mortality at 28 days

The 28-day mortality was higher in the non-sepsis group (34.5%) compared to the sepsis group (17.6%), (p = 0.023). The highest mortality rate was registered for patients with AKI-stage 3 in both groups, (Table 4). No significant difference in the median AKI stages was recorded among the deceased patients at 28-days in the sepsis versus the non-sepsis groups (Table 4).

**Table 4:** 28-day mortality in each AKI group and admittance values of hepcidin and HBP, respectively, in sepsis and non-sepsis patients. IQR, inter quartile range. The median AKI stage among sepsis patients was 3 (IQR 0–3) versus 2 (IQR 1–3) in non-sepsis patients that deceased within 28-days, calculated with Mann-Whitney U-test, (p = 0.639). AKI-stage 0 is equivalent to no kidney failure.

28-day mortality	Sepsis n = 15/85	Hepcidin nmol/L	HBP ng/mL	Non-sepsis n = 19/55	Hepcidin nmol/L	HBP ng/mL	p-value
AKI stage 0–3, median (IQR)	3 (0–3)	-	-	2 (1–3)	-	-	p = 0.639
AKI-0 (IQR)	5	31.0 (8.7–49.0)	34.6 (16.5–53.9)	4	6.8 (4.2–31.8)	13.5 (11.9–37.4)	
AKI-1 (IQR)	0	-	-	5	5.8 (2.1–35.5)	33.5 (22.5–83.2)	
AKI-2 (mean)	1	34.0	34.5	2	6.5	134.6	
AKI-3 (IQR)	9	39.0 (16.5–79.5)	69.3 (19.9–411.7)	8	27.5 (3.9–57.8)	38.2 (29.4–54.1)	

## Discussion

The clinical data presented in this study challenge the idea that serum hepcidin levels upon admission in intensive care patients serve as a marker for the development of acute kidney injury (AKI) [13]. Contrary to earlier reports, our findings do not align with the notion that elevated serum iron levels are linked to higher mortality in ICU patients with compromised kidney function [9].

This is further underlined by experimental studies in which administration of hepcidin reduced lipopolysaccharide, (LPS)-induced inflammation, reduced bacteraemia, and reduced AKI and mortality also in caecal-ligation peritonitis [45]. Furthermore, in a review and consensus document hepcidin was indeed suggested as a possible biomarker for the diagnosis and severity of AKI [46]. This was based on several studies including one that showed that low levels of urine hepcidin recorded at admittance to the ICU are associated with severe AKI, but do, however, not predict progression or severity of disease [47]. The patient cohort in the single centre study had AKI as an inclusion criterion [47]. Results from a multicentre study show that in a patient cohort in which RRT was inclusion criterion patients with plasma hepcidin levels in the lowest quartile ran a fourfold increased risk of death at 60 days [20]. A role of hepcidin in the development of AKI is also suggested by that urine hepcidin is significantly higher among patients who did not develop AKI after cardiopulmonary surgery [48] corroborated by a study by Prowle *et al*. who found urine hepcidin to be significantly higher in patients who did not develop AKI [25].

These earlier studies differ from the present in several important aspects. Here we investigated patients who were suffering from community-acquired sepsis or other severe conditions, and initial hepcidin levels were determined after admittance to the ICU within 24 hours after the initial admission to the hospital. We found no associations between hepcidin serum levels at admission with AKI-stage 2–3, RRT, or peak creatinine in the septic nor in the non-septic patient groups. Furthermore, the mortality after 28-days was not associated with low hepcidin levels at admission in the limited number of patients requiring RRT. Thus, our data do not support the hypothesis that increased serum hepcidin levels protected patients from developing AKI, need for RRT therapy, or survival at 28 days neither in the septic nor in the non-septic patient groups.

Levels of heparin-binding protein were significantly associated with AKI in our patients. Serum levels of HBP, have been shown to have a predictive potential as to development of AKI. Thus, HBP can discriminate patients who develop AKI stage 1–3 on day 2–5 of intensive care treatment from patients with a normal kidney function, and HBP is significantly associated with development of any AKI stage [30]. Here we found that septic patients to whom renal replacement therapy had to be administrated had higher serum levels of HBP at admission, whereas in the non-septic patients such a correlation was not found. This is in line with previous research, reporting the highest HBP levels among sepsis patients with AKI undergoing RRT [32]. A significant association between HBP serum concentration and peak creatinine was found only in the sepsis group. No significant association between HBP levels, peak creatinine, and the need for RRT was present among the non-septic patients in our cohort. Although a limited number of patients (n = 8) could be analysed it is possible that end-stage renal disease in fact is not associated with HBP in non-septic patients.

HBP can predict AKI stage 2–3 in a subgroup of ICU patients [31]. Pajenda *et al*. reported significantly higher mean values of HBP among sepsis patients with AKI compared to sepsis patients without AKI [32]. HBP adds a predictive value to other known risk factors for S-AKI, in 511 sepsis patients of whom 20% developed AKI stage 2–3 within 5 days [49].

Although our patient cohort was not directly comparable to abovementioned studies since our patient cohort included only community acquired critical illness, patient admitted to the ICU within 24 hours of hospitalisation, and exclusion of patients with chronic kidney disease, and erythrocyte transfusion prior to inclusion, the results presented in our study support the correlation between HBP and septic AKI. Furthermore, our study population was representative, reflecting the proportion of sepsis versus non-sepsis patients in the ICU, numbers developing AKI, and in need of RRT, along with the most common comorbidities [2,50].

Limitations of the present study were the rather small number of patients included in the cohort, including few patients in need of RRT, and that the classification of AKI depended on changes in creatinine levels compared to baseline, which is an unreliable method. Creatinine is known to be affected by many factors such as muscle mass, diet, and medication [51]. Almost a third of the patients in the present study had no baseline creatinine noted before admission, hence increasing the risk of over- or underestimating the AKI incidence. A large heterogenicity when defining serum creatinine is found in the literature. Missing baseline serum creatinine in reports on patient cohorts in the ICU range between 25–61% according to the literature, and thus our results can be considered representative [52,53]. Furthermore, creatinine only, was utilized to diagnose AKI in our cohort, since urinary output data was not reliable with missing data in patient records. The lack of urine output data is also a well-known problem according to earlier reports [43]. Even though patients were enrolled consecutively, another limitation was that not every eligible patient that arrived to the ICU was enrolled, due to workload and high time restraints in the ICU. Furthermore, confounding factors that may have inflicted the results of hepcidin levels was the fact that both genders had slight anaemia already at inclusion, (sepsis and non-sepsis group), and five of the sepsis patients had an underlying liver disease, in contrast to none in the non-sepsis group. We neither assessed mean corpuscular volume (MCV) nor the iron status in the investigated cohort, since it was not in scope of our study. Further research is warranted to explore the correlation between HBP and hepcidin, respectively, and with possibly better biomarkers for acute kidney injury such as cystatin C. Cystatin C is a biomarker known to have advantages over serum-creatinine [46,54].

## Conclusion

Hepcidin and HBP serum concentrations at admission to the ICU were significantly higher in sepsis patients compared to critically ill patients with non-infectious conditions treated in the ICU. In our cohort of patients with community acquired critical illness we could not demonstrate any significant associations between hepcidin levels at admission and stage 2–3 acute kidney injury, renal replacement therapy, or peak creatinine levels in neither sepsis nor non-sepsis patients.

On the contrary, HBP was significantly associated with acute kidney failure among critically ill patients both with and without sepsis. Furthermore, among the septic patients, there was an association between HBP and the need of renal replacement therapy as well as with peak creatinine. No significant correlation was demonstrated in the non-sepsis group either between HBP and peak creatinine or renal replacement therapy. The results of our limited study in ICU patients of a tertiary Swedish hospital show that admittance value of hepcidin is a significant marker of sepsis whereas HBP levels can predict renal failure in septic patients admitted to the ICU.

## Supporting information

S1 Data (SAV)
